# Supplementary material for: Calorie Restriction Upregulates Islet PD-L1 Signaling and Decreases the Risk of Autoimmune Diabetes Onset in NOD Mice
Source: bioRxiv. 2026 Feb 17:2026.02.15.705935. Preprint. [Version 1] doi: 10.64898/2026.02.15.705935 (PMC12934934; doi:10.64898/2026.02.15.705935)
Supplement: Supplement 1 — Supplementary Figure 1: (A) Lean mass and (B) fat mass. Data are presented as mean with 95% confidence interval (C.I.). A n = 24 AL mice and 25 CR mice. For two groups comparison, unpaired two-tailed Student’s t test was performed (A and B). All data presented as mean ±95% confidence intervals (C.I.). In (A-B) n= 15 AL and n= 15 CR mice per group. Supplementary Figure 2: (A) Blood glucose levels in NOD female mice under AL or CR over 2 months of dietary intervention, measured in fed and fasted states. (B) Probability of hyperglycemia onset over time in female NOD mice housed in the Vanderbilt animal facility. (C) Probability of hyperglycemia onset over time in fed or fasted female NOD mice under AL or CR over 2 months of dietary intervention. Survival was analyzed using Kaplan–Meier survival analysis with log-rank (Mantel–Cox) test (A-C). All data presented as mean ±95% confidence intervals (C.I.). In (A-C), data pooled from n=4 different cohorts, with a total of 59 AL and 53 CR mice at the start of the diet intervention. Supplementary Figure 3: (A) GSEA hallmarks for gene sets Hallmark_UNFOLDED_PROTEIN_RESPONSE. (B) GSEA hallmarks for gene sets Hallmark_MYC_TARGETS_V1. (C) GSEA hallmarks for gene sets Hallmark_MTORC1_SIGNALING. (D) GSEA hallmarks for gene sets Hallmark_COMPLEMENT. (E) GSEA hallmarks for gene sets Hallmark_P53_PATHWAY. (F) GSEA hallmarks for gene sets Hallmark_E2F_TARGETS. Supplementary Figure 4: (A) Quantification of insulitis area normalized to islet area in pancreatic sections from AL and CR female NOD mice after 2 months on diet. Each dot represents one islet. (B) Quantification of Ki67+ cells within the insulitis in AL and CR groups. Each dot represents one islet. (C) Relative distribution of T-cell populations in AL and CR female NOD mice, shown as stacked bar plots. T cells were classified into CD4 naïve, regulatory T cells (Treg), CD8 effector, proliferating T cells, and natural killer (NK) cells based on transcriptomic markers. Bar graphs d [file media-1.pdf]

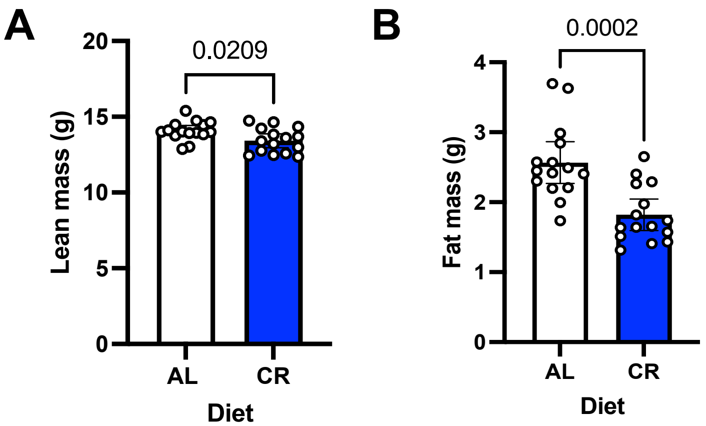

Supplementary Figure 1

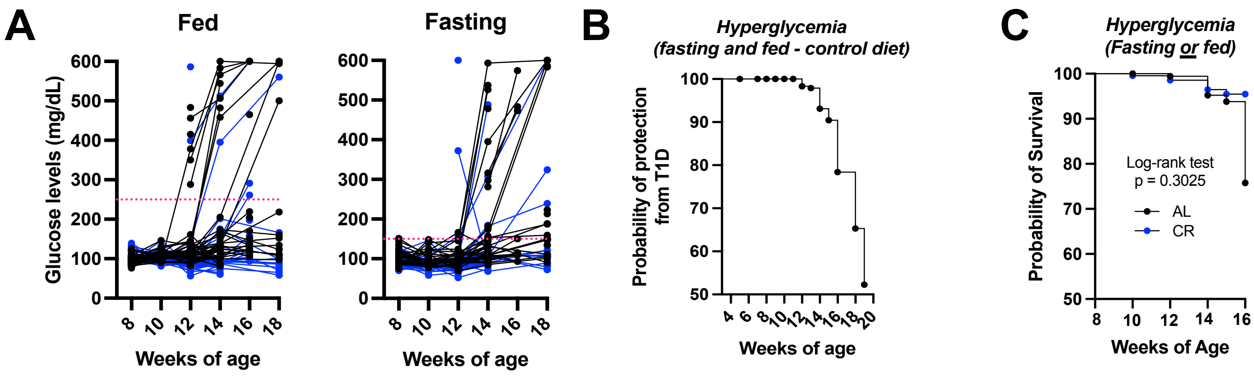

Supplementary Figure 2

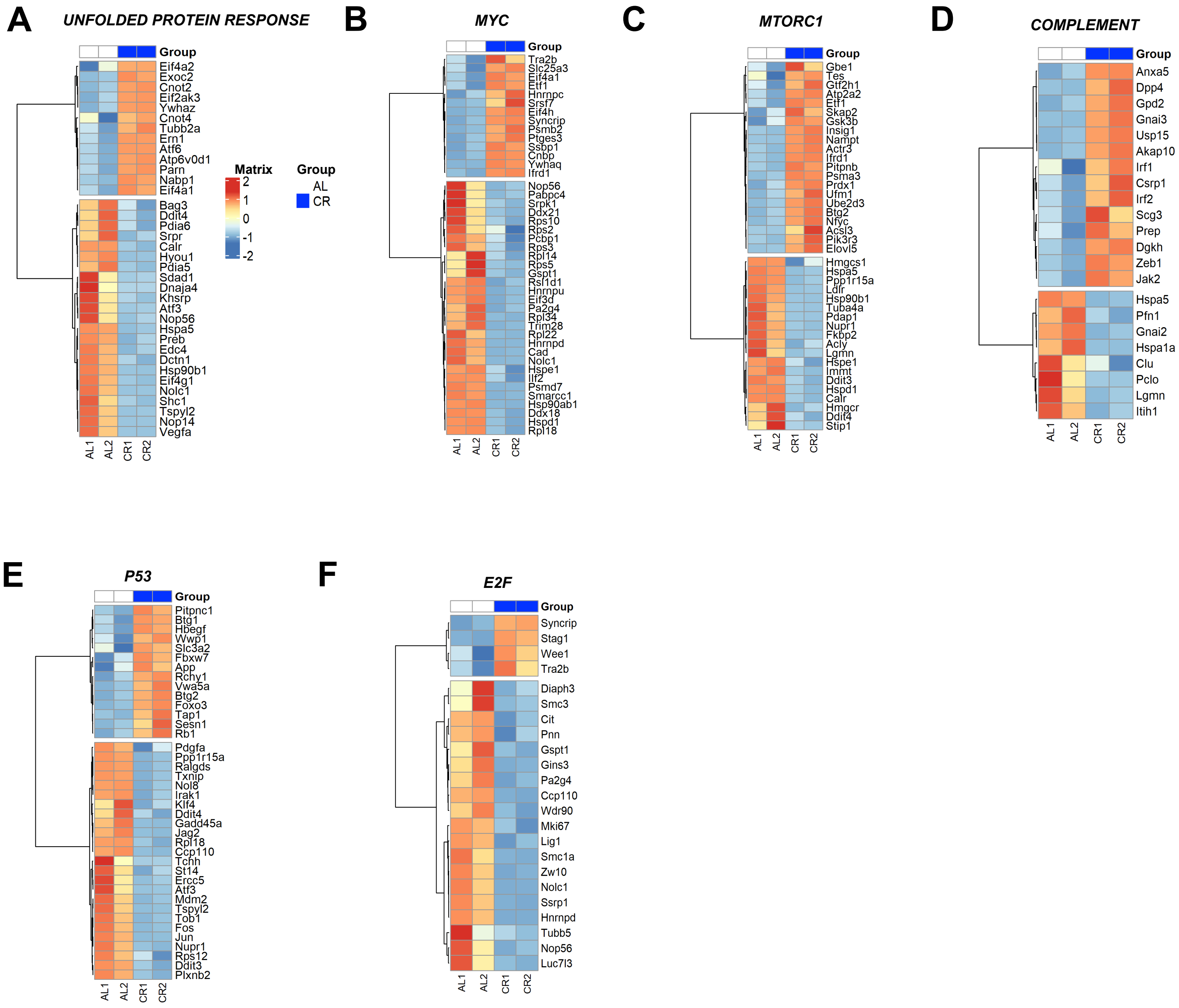

Supplementary Figure 3

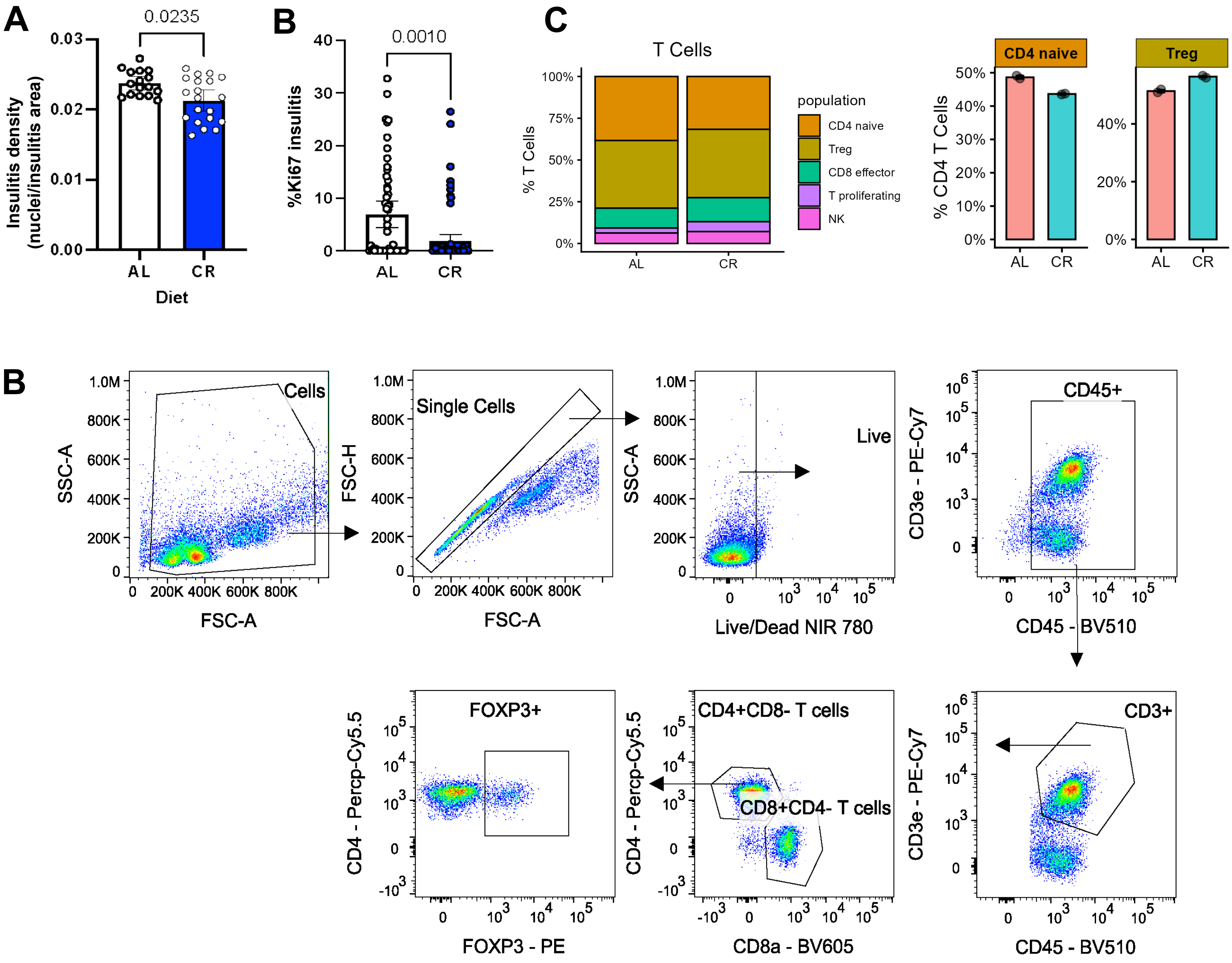

Supplementary Figure 4

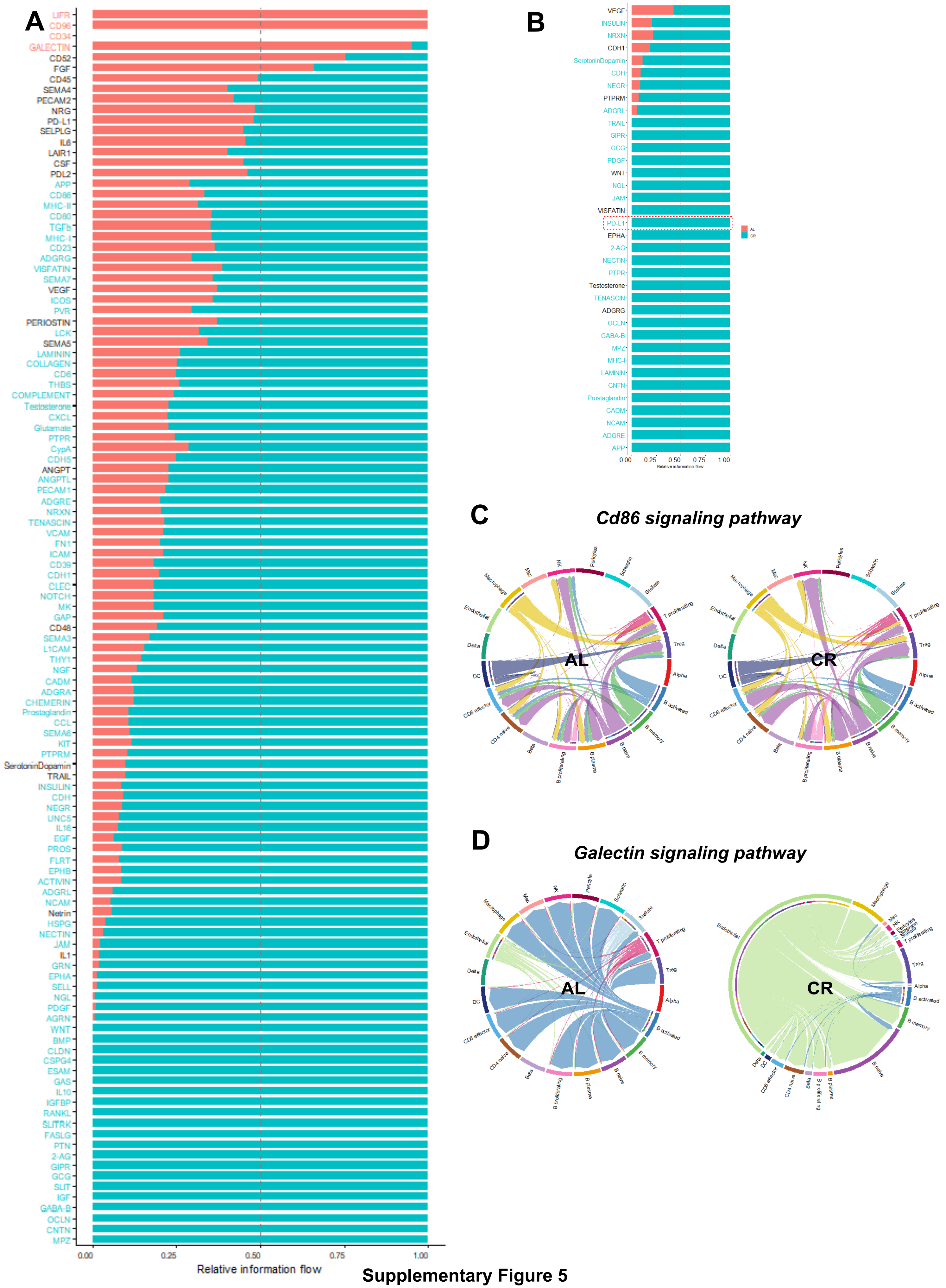

Supplementary Figure 5
