# Supplementary material for: Calorie Restriction Upregulates Islet PD-L1 Signaling and Decreases the Risk of Autoimmune Diabetes Onset in NOD Mice
Source: bioRxiv. 2026 Feb 17:2026.02.15.705935. Preprint. [Version 1] doi: 10.64898/2026.02.15.705935 (PMC12934934; doi:10.64898/2026.02.15.705935)
Supplement: Supplement 2 [file NIHPP2026.02.15.705935v1-supplement-2.pdf]

Supplementary Table Legends

Supplementary Table 1 – List of genes identified by GSEA analysis of AL and CR beta cells.

Supplementary Table 2 - List of genes identified by GSEA analysis of islet immune cell types in AL and CR mice.

Supplementary Table 3 – List of differently expressed genes in alpha cells.

Supplementary Table 4 – List of differently expressed genes in delta cells.

Supplementary Table 5 – List of exhaustion T cell markers for CD4 and CD8 cell types.
